# Uncommon but valuable for diagnosis: Green deposits on peritoneal fluid cytospins

**DOI:** 10.1002/ccr3.3834

**Published:** 2021-01-22

**Authors:** Jean‐François Lesesve, Delphine Gérard, Alice Burgevin

**Affiliations:** ^1^ Laboratory of Hematology University Hospital at Nancy Vandoeuvre France; ^2^ Department of Gastrointestinal Surgery University Hospital at Nancy Vandoeuvre France

**Keywords:** bile deposits, gallbladder perforation, morphology, peritoneal fluid

## Abstract

When ascites fluids are observed under the microscope, unshaped greenish deposits should not be considered as artifacts but should rather prompt to bile leakage assessment.

## Graphical abstract

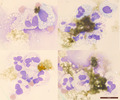

## 1 CASE HISTORY

Microscopic observations in the context of a gallbladder perforation are reported. Observation under the microscope of amorphous green deposits from the ascitic fluid lead to the diagnosis. Causes of green peritoneal fluid are reviewed.

A 37‐year‐old man without any history presented with abdominal pain. On examination, he manifested signs of generalized inflammation with suspected peritonitis. Laboratory studies showed leukocytosis 19 960/μL (88% neutrophils), sodium concentration 127 mmol/L, potassium 3.52 mmol/L, alkaline phosphatase 1049 IU/L (normal < 116), alanine transaminase 30 IU/L (<40), gamma‐glutamyl transferase 435 U/L (<73), total bilirubin 14 μmol/L (<21) (=8 mg/L, <12), ferritin 2,490 μ g/L (< 322), and C‐reactive protein 113 mg/L (<10). Computed tomography (CT) scan showed a necrotic area inside the pancreas concerning for acute pancreatitis. The gallbladder was not distended and was filled with uncomplicated stones without dilation of the bile ducts. After paracenteses of usual color for a few days, grossly greenish ascites appeared. A total of 0.4 liters of peritoneal fluid was drained and a sample sent to the laboratory for cytological examination.

**FIGURE 1:**
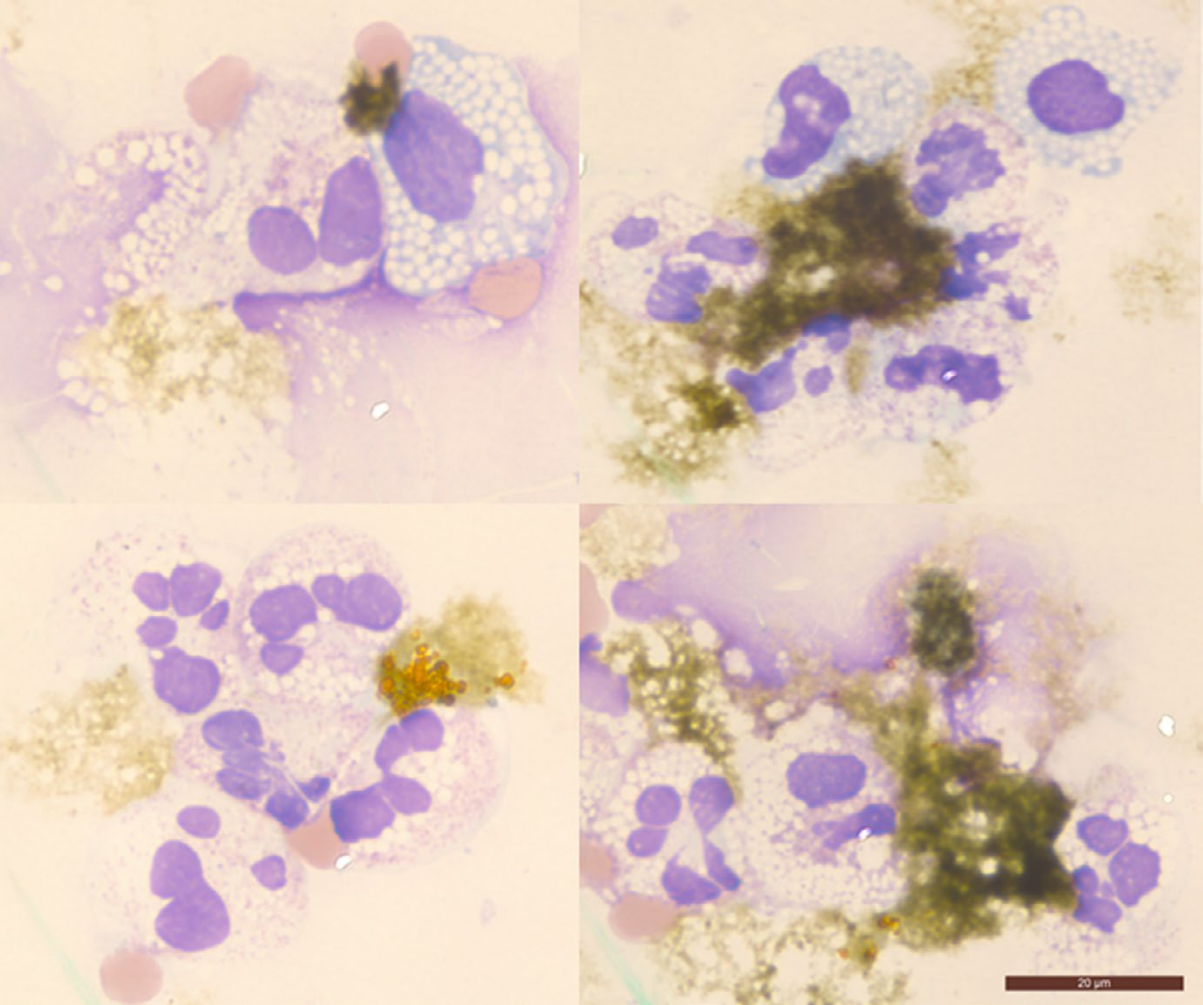
Ascites smears (May Grunwald Giemsa stain, ×1000). Scattered neutrophils showing mild nuclear atypia (hyper‐lobulation, myelokathexis) and vacuolated cytoplasm (all images) mixed with dystrophic monocytes (vacuolization, upper images). Green shapeless deposits of bile scattered outside the cells. Scale bar 20 μm

The liquid had a cloudy and slightly green appearance in the collection tube. Total mononuclear cells were 226/μL (91% neutrophils), proteins 15 g/L, albumin 7.6 g/L, glucose 4.40 mmol/L, triglycerides 0.72 mmol/L, amylase 18 IU/L, and bilirubin 11 mg/L. Cytospins showed an excess of dystrophic neutrophils and monocytes, as well as unusual and amorphous deposits, mainly characterized by their greenish color (Figure 1), raising the possibility of a bile leak. On review, the CT scan revealed a 2‐cm perforation in the inferior gallbladder. Adapted antibiotic therapy (piperacilline, cefepime, and metronidazole) was initiated after identification of *Enterobacter cloacae* + *Escherichia coli* in the ascites. Cholecystectomy and peritoneal lavage were performed. The patient recovered after several weeks of intensive care.

## 2 DISCUSSION

Gallbladder perforation is a rare but potentially lethal condition.^1^ It represents the most severe complication of cholecystis, mainly occurring in the context of an advanced inflammation, such as empyema or gangrene. Older men are predominantly affected by this condition, often in a context of neoplasia or steroid therapy.^2^, ^3^

The clinical diagnosis is often difficult and may be delayed. Typical symptoms include high fever, nausea, vomiting, and right upper quadrant abdominal pain.^4^ However, minimally symptomatic patients have also been reported. Differential diagnosis includes a spectrum of acute abdomen conditions, such as pancreatitis or peritonitis.^5^ Analysis of the ascitic fluid thus becomes a valuable tool, especially if a green color is macroscopically observed. Occasional bile pigment‐laden macrophages, extracellular lakes of amorphous green stringy material admixed with variable numbers of histiocytes, mesothelial cells, and acute and chronic inflammatory cells suggest the presence of bile^6^ and, consequently, a gallbladder perforation at first glimpse.^7^ Other bile stained ascitic fluid can result from complications of abdominal trauma, acute pancreatitis, or intestinal perforation.^8^ Other causes of green peritoneal fluid more rarely include hyperbilirubinemia and hemolysis after peritoneal hemorrhage. In remaining cases, the diagnosis of gallbladder perforation can be confirmed after radiological imaging (CT and/or magnetic resonance), bilirubin elevation above 6 mg/dL, ascitic fluid to serum bilirubin ratio greater than 1, and, most specifically, characterization of bile pigments through analysis by Raman or infrared microspectroscopy. Nonspecific cell dystrophies and lysis raise the concern for abdominal catastrophe. Therefore, the treatment should be straightforward, often consisting in a laparotomy/scopy followed by cholecystectomy/stomy. Unfortunately, the prognosis remains poor with high mortality.

As green peritoneal deposits may be associated with other viscera perforation, radiological investigation is essential to precisely make the diagnosis, even if the deposits might initially suggest bile deposits.

## 3 CONCLUSION

Appearance of green deposits in the peritoneal fluid warrants immediate investigation of the biliary tract, even for patients without abdominal symptoms.

## CONFLICT OF INTEREST

None declared.

## AUTHOR CONTRIBUTIONS

AB: followed the patients. JFL and DG: made microscopic investigations. JFL: wrote the manuscript.

## ETHICS, FUNDING AND DATA AVAILABILITY STATEMENT

Informed consent from the patient, data and films are available on demand. No funds were required for this report.
